# Rats Lacking the Dopamine Transporter Display Inflexibility in Innate and Learned Behavior

**DOI:** 10.3390/biomedicines12061270

**Published:** 2024-06-07

**Authors:** Anastasia Belskaya, Natalia Kurzina, Artem Savchenko, Ilya Sukhanov, Arina Gromova, Raul R. Gainetdinov, Anna Volnova

**Affiliations:** 1 Institute of Translational Biomedicine, Saint Petersburg State University, Saint Petersburg 199034, Russia; anastasia554@yandex.ru (A.B.);; 2 Valdman Institute of Pharmacology, Pavlov First St. Petersburg State Medical University, Saint Petersburg 197022, Russia; 3 Biological Faculty, Saint Petersburg State University, Saint Petersburg 199034, Russia; 4 Saint Petersburg University Hospital, Saint Petersburg 190121, Russia

**Keywords:** dopamine transporter, hyperdopaminergia, dopamine transporter knockout rodents, learning

## Abstract

Playing a key role in the organization of striatal motor output, the dopamine (DA)-ergic system regulates both innate and complex learned behaviors. Growing evidence clearly indicates the involvement of the DA-ergic system in different forms of repetitive (perseverative) behavior. Some of these behaviors accompany such disorders as obsessive–compulsive disorder (OCD), Tourette’s syndrome, schizophrenia, and addiction. In this study, we have traced how the inflexibility of repetitive reactions in the recently developed animal model of hyper-DA-ergia, dopamine transporter knockout rats (DAT-KO rats), affects the realization of innate behavior (grooming) and the learning of spatial (learning and reversal learning in T-maze) and non-spatial (extinction of operant reaction) tasks. We found that the microstructure of grooming in DAT-KO rats significantly differed in comparison to control rats. DAT-KO rats more often demonstrated a fixed syntactic chain, making fewer errors and very rarely missing the chain steps in comparison to control rats. DAT-KO rats’ behavior during inter-grooming intervals was completely different to the control animals. During learning and reversal learning in the T-maze, DAT-KO rats displayed pronounced patterns of hyperactivity and perseverative (stereotypical) activity, which led to worse learning and a worse performance of the task. Most of the DAT-KO rats could not properly learn the behavioral task in question. During re-learning, DAT-KO rats demonstrated rigid perseverative activity even in the absence of any reinforcement. In operant tasks, the mutant rats demonstrated poor extinction of operant lever pressing: they continued to perform lever presses despite no there being reinforcement. Our results suggest that abnormally elevated DA levels may be responsible for behavioral rigidity. It is conceivable that this phenomenon in DAT-KO rats reflects some of the behavioral traits observed in clinical conditions associated with endogenous or exogenous hyper-DA-ergia, such as schizophrenia, substance abuse, OCD, patients with Parkinson disease treated with DA mimetics, etc. Thus, DAT-KO rats may be a valuable behavioral model in the search for new pharmacological approaches to treat such illnesses.

## 1. Introduction

Dopamine (DA) is one of the main monoaminergic neurotransmitters in the central neural system. The DA-ergic system is critically involved in the control of many physiological functions and pathophysiological processes [1,2,3]. Extracellular DA neurotransmission mainly depends on the activity of the dopamine transporter (DAT), which provides this neurotransmitter’s homeostasis by transporting synaptic DA into the cytosol of the presynaptic neuron [4]. The impact of DAT’s contribution to DA turnover is especially high in the striatum, which contains ≈ 80% of this neurotransmitter within the brain [5,6].

Abnormalities in DA-ergic system functioning lead to the development of different neurological diseases [7,8]. In the search for and development of new therapeutic approaches for the treatment of these diseases, different animal models were created [9,10,11,12,13,14,15]. Rodents (mice and rats) with a knockout of the gene encoding the dopamine reuptake transporter protein (DAT-KO) are one of the most popular models of hyperdopaminergia [4,16,17,18]. A unique feature of this model is the increased extracellular DA levels in the brain. The DAT-KO rats are characterized by spontaneous hyperactivity, which is reduced via the administration of amphetamine or methylphenidate [18].

Playing a key role in the organization of striatal motor output, the DA-ergic system regulates both innate and complex learned behaviors [19]. Growing evidence clearly indicates the involvement of the DA system in different forms of repetitive behavior. For example, repetitive behaviors may be triggered in humans taking DA mimetics (e.g., amphetamine and L-DOPA) [20,21]. Disorders of the DA-ergic system seem to be involved in the pathogenesis of diseases with perseverations, such as obsessive–compulsive disorder (OCD), Tourette’s syndrome, schizophrenia, and addiction; for a review, see [22]. Analogically, the pharmacological administration of DA mimetics to mice and rats causes a wide spectrum of stereotype behaviors, including climbing, sniffing, licking, and biting [23,24]. The DAT-KO mice and rats also demonstrate some forms of repetitive motor behavior [25,26,27,28,29]. In this study, we would like to trace how the inflexibility of repetitive reactions in DAT-KO rats influences the realization of innate behavior and learning of spatial and non-spatial cognitive tasks.

## 2. Materials and Methods

### 2.1. Animals

Thirty-four DAT-KO and thirty-four WT control male rats of the same age (3–6 months) were used in the experiments (Experiment 1, 11 WT and 13 DAT-KO; Experiment 2, 10 WT and 10 DAT-KO; Experiment 3, 13 WT and 11 DAT-KO rats). The experiments were performed according to the regulations covering animal experimentation within the European Union (European Communities Council Directive 2010/69/EU), the Regulations on Research Using Experimental Animals (Order of Ministry of Health of the Russian Federation #742), the FELASA and RusLASA requirements regarding the care and treatment of laboratory animals, and with the requirements regarding the care and treatment of laboratory animals and the Ethics Committee of Saint Petersburg State University, St. Petersburg, Russia, resolution No. 131-03-10 of 22 November 2021. The experimental protocol of Experiment 3 was also approved by the local Animal Care and Use Committee of Pavlov Medical University (PMU), Russia.

Before the experiments, rats were maintained in IVC cages (RAIR IsoSystem World Cage 500; Lab Products, Inc., Aberdeen, MD, USA; Experiments 1 and 2) or in TIIIH (Tecniplast, Buguggiate, Italy, Experiment 3) cages with ad libitum access to filtered tap water and standard lab chow (formula ПK 120-1, Laboratorkorm, Moscow, Russia). Housing conditions included a temperature range of 22 ± 1 °C, relative humidity maintained between 50 and 70%, and a 12 h light/dark cycle (with light provided from 9 a.m. to 9 p.m.).

In Experiment 1, the animals had free access to food because this experiment did not involve learning. In Experiments 2 and 3, food intake was restricted before the start of the experiment, resulting in a decrease in their body weight to about 85% of their original weight. This was necessary to increase the animals’ motivation to learn and receive food reinforcement. During the experiments, the daily food allowance (15–16 g for WT rats and 20 g for DAT-KO rats) was regulated to limit body weight gain to 2–3 g per week. At the start of the experiments, the body weight of DAT-KO rats was 240 ± 10 g, lower than that of WT rats (310 ± 10 g). At the end of the experiments, the body weight of DAT-KO rats was 220 ± 10 g, and the average WT rat’s body weight was 280 ± 10 g.

The experiments were conducted between 2 p.m. and 6 p.m.

### 2.2. Experiment 1: Analysis of Grooming Behavior

To analyze grooming behavior, rats were placed in a transparent plastic box (25 cm × 25 cm × 25 cm). Every session continued for 20 min, and each rat was tested three days in a row. The testing boxes were cleaned with peroxide after every session. Autogrooming was videotaped by two side-view cameras. The videos were analyzed to assess autogrooming parameters such as the number of grooming bouts, mean bout duration, and the number of short episodes of autogrooming (less than 5 s). A new grooming bout started when the last grooming behavior terminated for more than 6 s [30].

The motor activity of animals in experimental boxes between episodes of autogrooming was also analyzed. Motor complexes consisting of running along the box wall plus rearings and semi-rearings, with head-raising in the corner of the box. These complexes were calculated. These manifestations (complexes) were observed in both control and knockout animals. The total number of complexes during the experiment and the dynamics of their variations during 20 min were compared.

To investigate the autogrooming microstructure, we evaluated the frequency of the occurrence of different stages in autogrooming [30]. The syntactic aspects of grooming were analyzed, forming fixed syntax patterns (chains). Syntactic grooming chains are conservative sequences that occasionally occur within grooming episodes [31,32]. Syntactic chains are composed of five consecutive phases. Phase 1 includes 5–9 rapid symmetrical forepaw strokes around the nose; phase 2 consists of 1–5 alternating unilateral paw strokes; phase 3 consists of bilateral and symmetric strokes of the head, usually extending past the ear; phase 4 is a rapid turn of the head towards the side of the body to commence licking of the body; phase 5 is the tail/genital grooming. The chain was considered to be initiated if the first stage was immediately followed by stages 2 or 3 [30]. To analyze the microstructure, we estimated the probability of syntax chain initiation as the frequency of fixed chain initiation (number of chains initiated divided by total grooming time). We also evaluated the total percentage of incorrect chains: the chains with returns and with the skipping of separate stages [30].

### 2.3. Experiment 2: Learning and Re-Learning in a T-Maze

The T-maze consists of a start zone (50 × 16 cm) and right and left arms (each 50 cm × 10 cm); the walls are 32 cm high. A rat approached the start zone through the guillotine door and then chose which arm to enter. Entries into both arms were open at the start of each trial. After the rat entered either of the arms, the guillotine door between the start zone and the arm zone was closed to prevent the rat returning to the start zone. Food reinforcement wells were placed at the end of each arm. Correct or incorrect choice arms were selected for each animal at random.

Stage 1: Learning. For every rat, we randomly selected an arm of the T-maze as the correct choice. At the end of that arm, we placed a food reward. For some rats, the reinforcement was placed in the right arm, for others it was placed in the left arm. Every animal made six runs in a row each day. After reaching the finish, the rat was placed in the home cage for 40 s before its next run. The maze was cleaned with peroxide after each animal’s trial to remove odor traces. At this stage of the experiment, rats had to learn that food was placed in a certain arm. Learning was considered successful when the animal made no less than five correct runs two days in a row (learning criteria). After learning was completed, re-learning started.

Stage 2: Re-learning. At this stage, we changed the test conditions for the rats that successfully learned the task. We switched the food location for each animal, so that the reinforcement was placed in the alternative arm. Re-learning was considered successful when the rat made no less than five correct runs two days in a row.

The dynamics of learning and re-learning in DAT-KO and WT rats (number of errors; number of days required to achieve the learning criteria) was analyzed. We videotaped the animal’s runs using a top-view camera; the choice time (time spent in the start zone) was measured and analyzed by a video tracking system (EthoVision XT, Noldus Information Technology, VA, USA).

### 2.4. Experiment 3: Extinction of Operant Responding

Six standard modular operant conditioning chambers for rats (30.5 cm × 24.1cm × 29.2 cm; ENV-007, MED Associates Inc., East Fairfeld, VT, USA) were used to study the operant behavior and Reilex production. The chambers were housed in light- and sound-proof ventilated boxes. Forty-five grams of food pellets (F0165, P.J. Noyes Inc., Lancaster, NH, USA) were used as the food reinforcement. A food pellet dispensing tray (ENV-200R2MA) equipped with infrared sensors (ENV254-CB) was placed 2 cm above the floor. Retractable levers (model ENV-112BM) were placed on the sides of the food tray. The chamber also had a speaker (ENV-223AM) and a white light (ENV-215M) at the top of the chamber. The methodology included the preliminary habituation of the animals to the experimental setup during the first training period (each rat was trained to consume food pellets). Rats were trained to receive food pellets after a sound signal. In the second training period, the fixed ratio schedules—one lever press (FR1), three lever presses (FR3) and five lever presses (FR5)—were reinforced by the delivery of one food pellet. The ratio was consistently increased from FR1 to FR3 and FR5. The details of the method were described previously [33]. In the first Progressive Ratio 3 (PR3) experimental design, the rat had to increase its force by three times in order to produce an increasing number of lever pressings (3, 6, 9, etc.) to receive the reinforcement (one food pellet). PR3 sessions lasted 120 min. The maximum ratio reached by the rat was recorded. Then, two days (and two FR5 sessions) after the PR3 experimental series, in the second experimental setup, rats were given an opportunity to press the lever without receiving any reinforcement. This made it possible to study the process of the extinction of the developed operant reaction (Ext schedule). The extinction session also lasted for 120 min. The following parameters were recorded: (1) the maximum ratio (MR) reached by rats during the PR3 schedule and Ext schedule; (2) time intervals between successive lever presses; (3) the percentage of rats that achieved the maximum ratio (MR) during the PR3 and Ext schedule.

### 2.5. Statistical Analysis

All results were analyzed using GraphPad Prism 8 (GraphPad Software, Inc., San Diego, CA, USA). The data were presented as the means ± SEM; *p* < 0.05 was considered statistically significant for all tests. A preliminary estimation of the data distribution normality (Gaussian distribution) was performed using the Kolmogorov–Smirnov test. We used the paired Mann–Whitney test (Experiments 1 and 2), the one-way ANOVA with the test for linear trends (Experiment 2), the one-way ANOVA test combined with the multiple comparisons post-hoc test (Experiment 3), the two-way ANOVA test combined with Sidak’s multiple comparisons post-hoc test (Experiments 2 and 3), and a comparison of survival curves via log-rank Mantel–Cox test (Experiments 2 and 3).

## 3. Results

### 3.1. Analysis of Grooming

**Figure 1:**
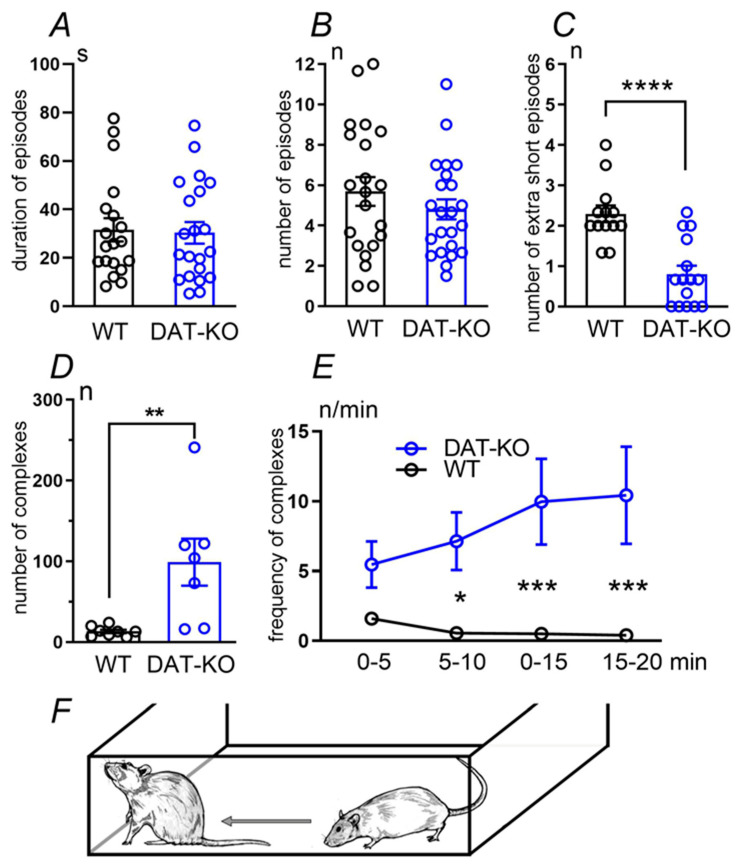
The general autogrooming analysis in WT and DAT-KO groups of rats. Analysis of episodes’ duration (**A**); the mean number of episodes (**B**) and the number of extra-short episodes (<5 s) (**C**); Analysis of fixed motor complexes as a manifestation of rat’s hyperactive behavior: the number of complexes (**D**); temporal dynamics: the frequency of complexes (number per min, **E**); the scheme illustrates what exactly was identified as a fixed motor complex (**F**). Results are presented as the mean ± SEM; **** *p* < 0.0001 **; *p* < 0.01, Mann–Whitney test (**C**,**D**); * *p* < 0.05, *** *p* < 0.001, two-way ANOVA test combined with Sidak’s multiple comparison post-hoc test (**E**).

In the first experiment, we examined general autogrooming parameters. The episode was considered autogrooming if it lasted over five seconds. The duration of episodes varied significantly: we observed both short and long episodes in both groups of animals. No significant differences in time spent on autogrooming were found (Figure 1A). The mean number of grooming episodes in DAT-KO or WT rats varied significantly (Figure 1B). However, ultra-short grooming, which is considered to be a stress-related or incomplete episode, was also identified (Figure 1C). This parameter differed significantly in both groups of animals (*p* < 0.0001 (Mann–Whitney test)).

The context of grooming behavior is very important for the evaluation of grooming distinctions in control and knockout rats. Although no significant differences in general autogrooming parameters were found in WT or DAT-KO rats, the behavior of these animals during the testing period differed dramatically. WT rats tended to calm down after the initial exploration of the box. Between episodes of autogrooming, they were quiet and showed mild motor activity in the experimental box. In contrast, DAT-KO rats showed pronounced motor hyperactivity.

To assess hyperactive behavior and identify its characteristic features, we analyzed fixed motor complexes consisting of a rat running along the wall accompanied by rearings or head raising in the corner of the box (Figure 1D–F). This type of behavior was found in both WT rats and knockout animals. A comparative analysis showed that the number of these motor complexes was significantly greater in DAT-KO rats than in WT animals (Figure 1D). An analysis of the dynamics of the number of these complexes indicates that, in WT rats, their frequency significantly decreased during the experiment. At the same time, in DAT-KO rats, the frequency of these behavioral acts not only did not decrease, but increased (Figure 1E). A statistical analysis confirmed that these differences were significant (two-way ANOVA, factor “group”, *p* < 0.0001). There were also significant differences in the frequency of complexes in the second (*p* < 0.05), third, and final (*p* < 0.001) five-minute time intervals of the experiment (two-way ANOVA test with Sidak’s multiple comparisons post-hoc test).

Thus, the data obtained indicate differences in the intergrooming behavior in the experimental box and some general parameters of autogrooming in DAT-KO and WT rats. However, more significant differences were revealed in the microstructure of autogrooming.

**Figure 2:**
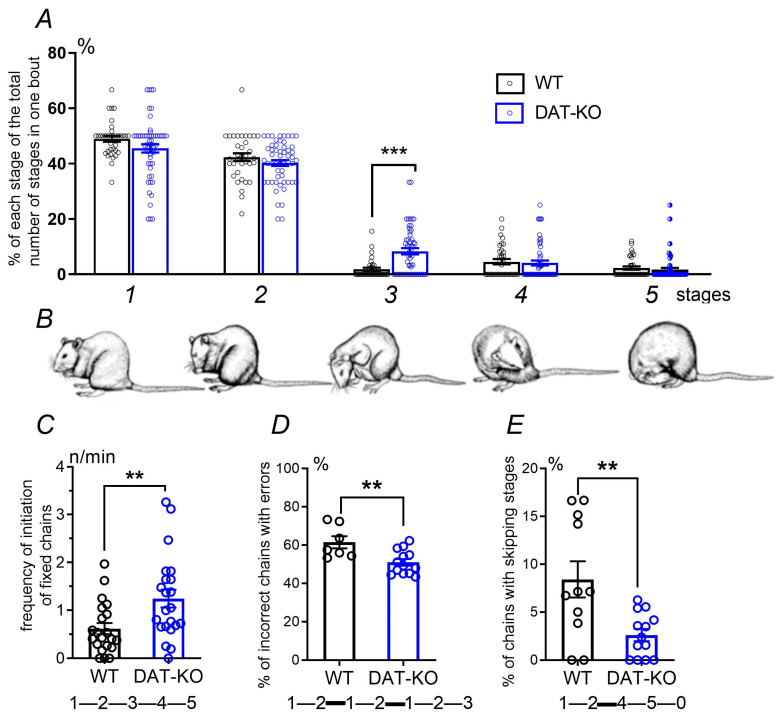
Analysis of the microstructure of autogrooming in the WT and DAT-KO groups of rats. The stages of autogrooming bouts in rats: number of occurrences of certain stages divided by the general number of stages in one bout, in % (**A**); results are presented as the mean ± SEM; ***—*p* < 0.001; Mann–Whitney test. Analysis of the microstructure of autogrooming: frequency of initiation of fixed chains (**C**); the percentage of incorrect chains with returns (**D**); the percentage of chains with skipping of separate stages (**E**). The numbers below the diagrams correspond to the stages of grooming (**B**); examples of fixed chains are presented; erroneous transitions between autogrooming stages are highlighted with bold lines; results are presented as the mean ± SEM; ** *p* < 0.01, Mann–Whitney test.

In accordance with previous investigations [30,31], the stages of autogrooming were identified from the rostral to the caudal direction: 1—paw licking; 2—face washing; 3—head washing; 4—body washing; 5—tail and genital washing (Figure 2A,B). The frequency of occurrence of each step in the autogrooming episodes in the two groups of animals was analyzed. It was found that only the third stage of autogrooming (head washing) underwent significant changes. Knockout rats demonstrated this stage of autogrooming more often than WT rats (*p* < 0.001; Mann–Whitney test).

A detailed analysis of the autogrooming microstructure revealed significant differences between the animal groups across various parameters. First, we estimated the probability of the occurrence of a fixed syntactic chain by dividing the number of such chains by the total grooming time [34]. It was found that DAT-KO rats tended to demonstrate such syntactic sequences more frequently compared to wild-type animals (Figure 2C). This observation is consistent with the fact that, in DAT-KO rats, episodes of extra-short autogrooming were observed significantly less often (Figure 1C). Next, errors in syntax and deviations from the correct order of stages during auto-grooming were analyzed. DAT-KO rats made significantly fewer errors in the sequence of fixed chain stages. This means that they had a significantly lower percentage of syntactic chains with errors (Figure 2D) and fewer chains with missing steps (Figure 2E). The obtained data indicate a high level of motor rigidity in rats lacking the dopamine transporter gene.

### 3.2. T-Maze

In Experiment 2, we compared the ability of knockout and wild-type rats to learn and relearn in a T-shaped maze. Note that, during learning, WT rats demonstrated a decrease in the number of errors, whereas some knockout rats showed perseverative behavioral patterns and consistently ran into the arm that contained a reward in the first experiment. They did not find any food there, but persistently ran into the unrewarded arm.

**Figure 3:**
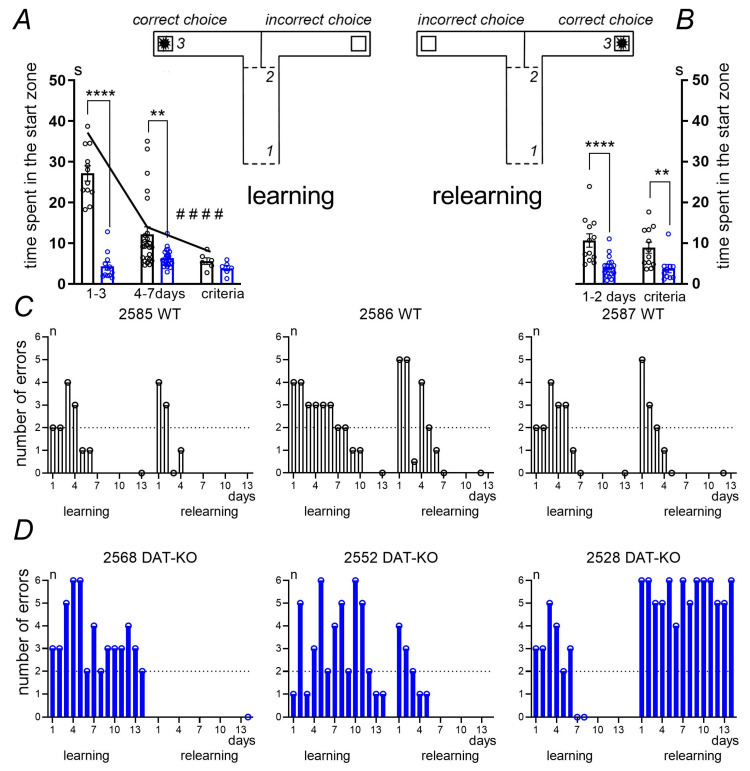
The analysis of the learning and re-learning of WT and DAT-KO rats in a T-maze. T-maze used in the experiment: learning stage (**A**) and re-learning stage (**B**), 1 and 2—guillotine doors; 3—food reward. Correct or incorrect choice arms were selected for each animal at random. The time spent in the start zone (in seconds); learning (**A**): 1–3 days, 4–7 days, days to reach the criteria; re-learning (**B**): 1–2 days, from the 3rd day to the day of reaching the criteria; results are presented as the mean ± SEM; ****—*p* < 0.0001; **—*p* < 0.01; Mann–Whitney test; #### *p* < 0.0001—ordinary one-way ANOVA, test for linear trend. Examples of learning and re-learning dynamics in WT (**C**) and DAT-KO (**D**) rats; individual rat numbers are indicated above the diagrams; WT—wild type, KO—knockout rats; the abscissa, days of experiments; the ordinates, the number of errors in the choice of the reinforced arm; the dashed line indicates the level of fulfillment of the learning criterion (less than two erroneous choices).

To analyze the exploration strategy, we compared the time spent by WT and DAT-KO rats in the maze start zone, considering this as the time the rat needed to choose which arm to run to. (Figure 3A,B). In the first three days of the learning experiment, WT rats spent significantly more time in the start zone than DAT-KO rats (*p* < 0.0001; Mann–Whitney test). During further training (learning), WT rats tended to progressively spend less time in the start zone, showing a significant decrease in the decision-making time for choosing the correct maze arm (#### *p* < 0.0001—ordinary one-way ANOVA, test for linear trend, Figure 3A). In the re-learning experiment, the time WT rats spent in the start zone increased again due the changed conditions (Figure 3B). In comparison with WT rats, DAT-KO rats spent equally short periods of time in the start zone during both training and re-learning. It can be supposed that the pronounced motor hyperactivity of DAT-KO rats prevents them from spending enough time to ensure correct decision-making regarding their run direction. For this reason, the number of erroneous trials increases and learning efficiency decreases.

Figure 3C,D illustrate the peculiar properties of learning and re-learning in individual WT and DAT-KO rats. All WT rats (for example, 2586 WT, 2587 WT, and 2585 WT) were able to learn and re-learn task rules during both stages of the experiments (Figure 3C). Some DAT-KO rats (for example, 2568 KO) failed to learn the task at stage 1 of the experiment (learning); however, another animal (2528 KO) was able to learn the task at stage 1 but failed to re-learn the task. This animal continued to select the maze arm in which the food reinforcement was in stage 1. Only a few animals (for example, rat 2552 KO) were able to both learn and re-learn, but they took a longer time to fulfil the learning criteria (Figure 3D).

**Figure 4:**
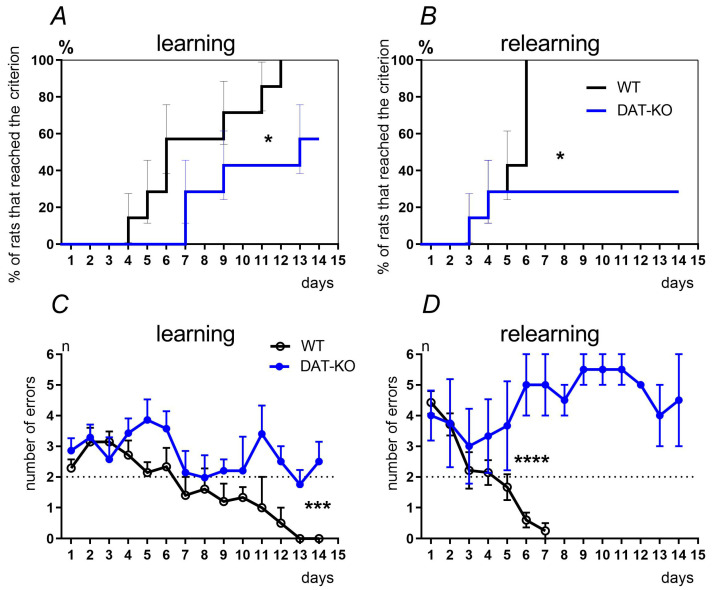
The dynamics of learning and re-learning of WT and DAT-KO rats in the T-maze. Number of animals (in percentage) who reached the criterion (less than two errors) during learning (**A**) and re-learning (**B**); the abscissa, days of the experiments; the ordinate, the percentage of rats who reached the <2 error criterion on a particular day of the experiment; comparison of “survival” curves using the log-rank (Mantel–Cox) test, *—*p* < 0.05. The dynamics of the number of errors in the choice of the reinforced arm during learning (**C**) and re-learning (**D**) in the T-maze; the abscissa, experimental days; the ordinates, the number of errors, where the dashed line indicates the level of fulfillment of the learning criterion (less than two erroneous choices); results are presented as the mean ± SEM, *** *p* < 0.001; **** *p* < 0.0001; two-way ANOVA test by the factor “groups”.

The abilities of WT and DAT-KO rats to learn and re-learn spatial discriminative tasks in the T-maze were compared (Figure 4A,B). All WT rats (100%) were able to solve the task, while only 57.2% of DAT-KO rats fulfilled the learning criteria (Figure 4A). During stage 2 of the experiments (re-learning in T-maze), all WT rats (100%) were able to switch the runs to the rewarded arm of the maze. Only 28.6% of DAT-KO rats were able to learn the new task rules (Figure 4B). Other DAT-KO animals visited the arms randomly and did not change their behaviors in accordance with the reinforcement presence. A comparison of the results revealed significant differences between the groups of WT and DAT-KO rats: log-rank (Mantel–Cox) test, *p* = 0.0218 for learning, and *p* = 0.0415 for re-learning.

The dynamics of the learning and re-learning of WT and DAT-KO rats were evaluated (Figure 4C,D). At stage 1 of the experiment (learning), WT rats fulfilled the criterion, on average, by the 6th day (Figure 4C), while stage 2 (re-learning) took them an average of 5 days (Figure 4D). DAT-KO rats exhibited significantly worse learning and re-learning dynamics, which were significantly different from those of wild-type animals (*** *p* < 0.001; F (1, 120) = 15.91; **** *p* < 0.0001; F (1, 51) = 19.72; two-way ANOVA test by the factor “groups”). This fact might also account for the inability of most knockout rats that were previously capable of reaching the learning criterion at stage 1 to choose the reinforced maze arm under changed task conditions.

### 3.3. Extinction of Operant Behavior

**Figure 5:**
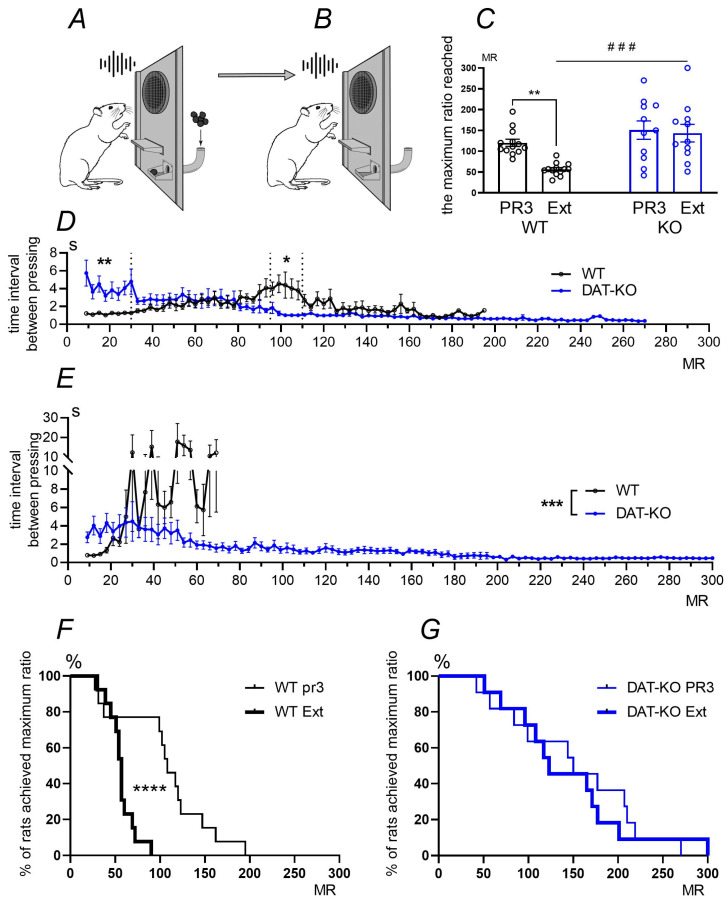
A study of operant behavior. The scheme of the experiment; the schedule of food reinforcement with a progressive ratio 3, PR3 (**A**), and the extinction of the learned reaction without reinforcement, Ext (**B**). The maximum ratio (MR) reached by rats during the PR3 schedule and Ext schedule (**C**); results are presented as the mean ± SEM. ### *p* < 0.001; ** *p* < 0.01; one-way ANOVA test combined with Sidak’s multiple comparisons post-hoc test. The time interval between lever pressing by rats during PR3 schedule (**D**) and Ext schedule (**E**). MR—the maximum ratio reached by rats; * *p* < 0.05; ** *p* < 0.01; *** *p* < 0.001; two-way ANOVA test combined with Sidak’s multiple comparison post-hoc test. The number of WT (**F**) and DAT-KO (**G**) rats (in percentage) that achieved the maximum ratio (MR) during the PR3 schedule (thin line) and Ext schedule (thick line). Comparison of survival curves using log-rank (Mantel-Cox) test between PR3 and Ext in WT rats: ****—*p* < 0.0001.

In Experiment 3, we examined the operant behavior using an operant schedule of food reinforcement with a progressive ratio of 3 (PR3) (the ratio increases by 3 after each reinforcement, as shown in Figure 5A). This was followed by the extinction of the learned reaction in an experimental setup (extinction schedule, Ext, Figure 5B). In this case, rats pressed a lever without receiving any reinforcement.

It was shown that in WT and DAT-KO rats with the PR3 schedule, the mean values of the maximum ratio (MR) of lever pressing for reinforcement were not significantly different (Figure 5C). However, after switching to the Ext schedule, the control group rats significantly reduced their efforts because they did not receive a reinforcement (** *p* < 0.01; one-way ANOVA test, Sidak’s post-hoc test); no such reduction in MR was observed in the knockout animals, as shown in Figure 5C. The comparison of control and knockout rats showed that DAT-KO rats achieved a significantly higher maximum ratio at the Ext schedule than WT rats (### *p* < 0.001; one-way ANOVA test, Sidak’s post-hoc test).

To reveal the dynamics of operant behavior under different schedules, the time intervals between lever pressing by WT and DAT-KO rats during the PR3 schedule (Figure 5D) and Ext schedule (Figure 5E) were analyzed. When performing operant behavior under the PR3 schedule, WT rats gradually increased the time interval between pressings, whereas the efforts required to obtain reinforcements increased. In the final period of the experiment, this parameter slightly decreased (Figure 5D). In contrast to the control group, DAT-KO rats demonstrated longer time intervals (4 s on average) between pressings at the initial stage. Thus, as MR increased, this parameter decreased and remained at its minimum level until the end of the PR3 schedule. During the extinction schedule (Ext), rats could press a lever but received no reinforcement (Figure 5E). The wild-type rats increased the interval between lever pressings quite rapidly to 10–15 s, and then stopped because they did not receive any reinforcement. Surprisingly, DAT-KO rats continued to press the lever, gradually decreasing the interval between pressings, despite the absence of any reinforcement.

We also analyzed the number of WT and DAT-KO rats that achieved the maximum ratio (MR) during PR3 and Ext schedules using a survival curve analysis that reflects the percentage of rats that reached a given MR (Figure 5F,G). The comparison showed that when changing the experimental setup from a PR3 to Ext schedule, WT rats saved a lot of effort; the number of rats that reached MR = 50 sharply decreased, and only a small number of animals reached MR = 90 (*p* < 0.0001) (Figure 5F). In contrast, DAT-KO rats did not change their behavior, and even in the absence of a reinforcement, a large number of animals achieved MR = 50 and some reached MR = 300 (Figure 5G). There were no significant differences between the implementation of behavioral tactics in PR3 and Ext schedules in the knockout group.

## 4. Discussion

The key role of DAT in the development of certain DA-related pathologies has been described in many studies [13,35,36,37,38,39,40,41]. DAT-KO rats are characterized by a specific set of behavioral deficits, such as hyperactivity, impulsivity, and inattention. In this study, we compared the innate and learned forms of behavior in DAT-KO rats, with a focus on perseverative reactions. According to our results, the silencing of DAT leads to behavioral inflexibility in rats. We demonstrated a high level of motor rigidity in autogrooming, perseverative patterns, and incorrect repeated runs in the T-maze, as well as resistance to the extinction of operant responses, in DAT-KO rats.

Grooming sequences represent innate motor stereotypes, which animals perform uniformly in a permanent order [30,42] and DA is a crucial neurotransmitter for the implementation of sequential grooming patterns [43]. It has been found that disruptions in the grooming chain may be found in animal models of DA-related diseases such as Huntington’s disease [44] and Tourette’s syndrome [45,46,47]. It was shown that elevated DA levels in transgenic mice induced super-stereotypy, which manifested itself in the form of the exceedingly rigid execution of syntactic grooming chains [32]. At the same time, the mutant mice without D1 receptors were not able to complete the grooming chains [48]. Interestingly, it was proposed that the excessive repetition of grooming chains in D1 agonist-treated rats can provide a potential model for the complex tics observed in Tourette’s syndrome and obsessive–compulsive disorder (OCD) [49].

In our experiments, the total time spent on autogrooming during the session did not significantly differ across the groups. The main differences between WT and DAT-KO rats were found in the autogrooming microstructure. The probability of syntactic chain occurrence was significantly higher in DAT-KO rats, and they made fewer errors during fixed chain realization. These findings indicate that DAT-KO rats maintain their grooming stereotypy and perform grooming chains in a more rigid manner than control animals. These findings coincide with the super-stereotypy revealed in the transgenic mice with a lack of DAT [32]. These behavioral patterns, to some extent, may mimic the manifestations of Tourette’s syndrome and OCD. It is necessary to mention that the intergrooming behavior in DAT-KO rats dramatically differs from WT rats’ behavior and is characterized by a high level of hyperactivity, while control animals showed no hyperactive reactions. We suppose that hyperactivity and stereotypy may have reciprocal relationships and strengthen each other.

DAT-KO rats are found to show impairments, not only in innate but also in learned behavior [39,40]. In experiment 2, we tried to train DAT-KO rats to follow simple rules (turning to the certain arm) in a T-maze to earn food reinforcement. We found that only half of the of DAT-KO rats were able to learn and perform the task in comparison with WT conspecifics when training to complete the simple task in the T-maze. Pronounced perseverative activity was also clearly demonstrated in the case of reversal learning. Only 28.6% of DAT-KO rats reached the learning criteria during this phase of experiment 2. Most of the DAT-KO rats constantly ran to the previously reinforced arm and did not choose the new correct one. The abnormal learning and re-learning of this behavioral task in a T-maze may be connected to the impairment of spatial memory in these animals, as described in our experiments [39].

Different neuromorphological changes were found in the brain areas of animals with DAT hypofunction. For example, dopamine transporter knock-down mice have fewer DA neurons in the substantia nigra from birth to aging [50]. A significant decrease in the spine density of pyramidal neurons was found in the mPFC and the CA1 regions of the hippocampus in DAT-KO mice [51]. In DAT-KO rats, a decrease in the volume of the dorsal striatum, accompanied by a distinct increase in the volume of the prefrontal and motor cortex, thalamus, and cerebellum, was described [52]. It was proposed that these brain changes can contribute to the impaired control of motor inhibition and hyperactivity. We suppose that, in terms of innate behavior, the perseverative reactions are provided by the striatum, whereas, in learned behavior, the cortical structures may be implicated. Thus, motor hyperactivity and perseverative responses in DAT-KO rats may be partly due to morphologic changes in the DA-ergic brain areas.

One of the potential explanations for this perseverative activity during reversal learning is tolerance to extinction. Previously, it was found that, in mice, DAT silencing led to enhanced resistance to the extinction of food-reinforced operant nosepoking [53] or amphetamine-induced conditioned place preference [54]. In Experiment 3, we presented a similar task (operant schedule “progressive ratio 3”) to DAT-KO rats. First of all, in accordance with our previous results [12], the DAT-KO rats demonstrated a changed pattern of lever pressing in comparison with the WT rats. The low FRs time between lever pressings was longer in DAT-KO than in WT rats, at a high FR, conversely, knockout rodents performed lever pressings more frequently and the interval between pressings became shorter than in control animals. During the extinction session, WT rats stopped performing the task significantly faster than DAT-KO rats, who continued to press the lever even in the absence of food. Thus, DAT-KO rats continued to repeat the previously learned skill despite the fact that pressing the lever did not result in reinforcement.

In general, the data of the present study demonstrate the low level of flexibility in innate and learned behaviors in rats with the silencing of DAT. The revealed inflexibility may reflect decreased stimulus control in the DAT-KO rats’ behavior. Our findings are in concordance with some previous studies that reported perseverative patterns of behavior in animals with a lack of DAT. Therefore, the hyperlocomotion of DAT-KO mice and rats is characterized by non-focal preservative patterns [29,52,55,56]. According to Rodriguiz and colleagues’ study of social behavior in DAT-KO mice, these animals demonstrated repetitive behavioral sequences [57]. Also, rigid choices and compulsive stereotypes in DAT-KO rats were reported by Cinque et al. [25]. The DAT-KO rats also demonstrated differences in the motivational and consummatory aspects of sexual behavior in comparison with WT rats [58]. They had a higher level of sexual activity and needed fewer copulatory tests to achieve a stable level of sexual activity than WT rats. This fact may be connected with knockout animals’ tendency towards stereotypy and repeating different types of behavior.

Based on a review of our own and the literature data, it is conceivable that the behavioral inflexibility demonstrated in rats with DAT silencing may reflect some of the behavioral traits observed in clinical conditions associated with endogenous or exogenous hyper-DA-ergia, such as schizophrenia, substance abuse, OCD, and other conditions [22,59,60]. For example, chronic L-DOPA treatment of patients with Parkinson disease (PD), in our opinion, fairly accurately reflects persistent hyper-Da-ergia in rodents with the silencing of DAT. It is known that compulsive behavior patterns develop in 13–24% of PD patients [61,62,63] and include a spectrum of behavioral abnormalities, according to Holden [64]—including, but not exclusively, pathological gambling, hypersexuality, binge eating, trichotillomania, kleptomania, and compulsive buying [65,66,67,68]. These repetitive or compulsive behaviors and decreased control [65,67] seem to be very close to our observations in DAT-KO rats.

## 5. Conclusions

From this point of view, DAT-KO rats can be used as model animals for the development of pharmacological approaches to control aberrant behaviors in patients with compulsivity, OCD traits, and side effects of PD treatment. All these data lead us to think that DAT-KO rats may be valuable not only as a model of hyperactivity in ADHD, but also in the study of other neurological diseases, such as OCD, Tourette’s syndrome, and even PD.

## Data Availability

The raw data used in this study are available on request from the corresponding author.
